# Psychological capital and music performance anxiety: the mediating role of self-esteem and flow experience

**DOI:** 10.3389/fpsyg.2024.1461235

**Published:** 2024-09-13

**Authors:** Xin Jiang, Yanli Tong

**Affiliations:** ^1^Department of Music, Universiti Malaya, Kuala Lumpur, Malaysia; ^2^Faculty of Language and Culture, Ningde Normal University, Ningde, China

**Keywords:** music performance anxiety, psychological capital, flow experience, self-esteem, music major undergraduates

## Abstract

**Background:**

This study aimed to explore the correlation between psychological capital and music performance anxiety among Chinese college students majoring in music. Additionally, the study evaluated the chain mediating effects of self-esteem and flow experience.

**Methods:**

Three hundred twenty-nine college students pursuing a music major from institutions in Eastern China participated in the study. They were asked to complete the Psychological Capital, Self-Esteem, Flow Experience, and Music Performance Anxiety Scales. Descriptive statistics and correlation analysis were performed, and sophisticated mediation models were created.

**Results:**

The results suggest that self-esteem and flow experience play a role in connecting psychological capital and music performance anxiety. Moreover, the ultimate model elucidates significant connections linking psychological capital to self-esteem and flow experience in music performance anxiety.

**Conclusion:**

This study offers useful insights for future interventions aimed at mitigating music performance anxiety through the cultivation of students’ psychological capital, self-esteem, and flow experiences.

## 1 Introduction

Mental health factors are considered one of the potential contributors to music performance anxiety (MPA). Prior research has found that depression is a significant predictor of MPA severity (Barbar et al., 2014; Kenny et al., 2014; Robson and Kenny, 2017). According to Bannai et al. (2016), people with symptoms of depression are more susceptible to adverse physical and psychological reactions during music concerts. Holding excessively rigid standards can cause musicians to internalize a great deal of pressure, and the relentless pursuit of perfection can increase performance anxiety (Yöndem, 2012). Additionally, it has been determined that both positive and negative self-concepts exert a significant influence on MPA. Those with a positive self-concept experience lower levels of MPA (Wiedemann et al., 2020). Music performance anxiety (MPA) refers to a distinct type of emotional behavior that is believed to be linked to a specific physiological state associated with anxiety (Altenmüller et al., 2012; Kenny, 2011). Common symptoms of MPA typically encompass accelerated heart rate, perspiration, and difficulty breathing, among others (Wesner et al., 1990). Research has indicated that anxiety related to performing is one of the deleterious elements that significantly impact artists’ and students’ careers in music performance (Fehm and Schmidt, 2006). This pertains to both adult musicians and performers as well as teenagers and children who are learning music (Papageorgi, 2022; Patston and Osborne, 2016; Urruzola and Bernaras, 2020). While a low level of MPA might enhance performance quality (Osborne and Franklin, 2002), extreme MPA can lead to significant outcomes, such as abandoning a career in music performance and adversely impacting the health and well-being of artists (Patston and Osborne, 2016).

Many factors influence music performance anxiety. Wilson and Roland (2002) introduced a three-dimensional framework to elucidate that music performance anxiety is primarily influenced by three factors (trait anxiety): individual attributes, surroundings, and past encounters. Prior research has indicated that music performance anxiety is impacted by age and gender. Students who start learning musical instruments at an earlier age generally show reduced levels of music performance anxiety (Dempsey and Comeau, 2019; Lupiáñez et al., 2022; Zarza-Alzugaray et al., 2018). Multiple studies have consistently found that female performers demonstrate higher levels of MPA compared to males (Butković et al., 2022; Dobos et al., 2019; Iusca and Dafinoiu, 2012; Orejudo et al., 2017; Sokoli et al., 2022). Chanwimalueang et al. (2017) suggested that this outcome might be explained by the heightened activation of the parasympathetic nervous system in females during the transition from pre-performance to performance. Environmental and situational circumstances are another important factor affecting music performance anxiety. Studies have shown that individual performances are associated with higher levels of MPA than group performances, and that musicians experience higher MPA levels during concerts compared to daily rehearsals (Cox and Kenardy, 1993; Orejudo et al., 2017; Studer et al., 2012). Papageorgi et al. (2013) suggested that the working environment of Western classical musicians may contribute to increased stress and anxiety levels. Students’ perceptions and strategies for learning and performing music are greatly impacted by the learning environment (Papageorgi et al., 2010). Simultaneously, the music learning environment also affects students’ motivation and levels of music performance anxiety (Casanova et al., 2018; Zarza et al., 2016). Furthermore, extensive performance experience and formal training contribute to lower levels of music performance anxiety (Coşkun-Şentürk and Çırakoğlu, 2018; Paliaukiene et al., 2018). However, Lupiáñez et al. (2022) discovered a positive relationship between MPA and music performance experience, indicating that experienced music performers are more likely to suffer higher levels of MPA. For this reason, providing focused intervention strategies for musicians to manage anxiety related to performing during the music performance learning stage is essential (Osborne and Kenny, 2008).

Personal factors are predictors of performance anxiety in music learners (Papageorgi, 2022). Research has consistently indicated that those with low self-confidence (Barbar et al., 2014; Miller and Chesky, 2004) and high perfectionism (Butković et al., 2022; Dobos et al., 2019; Liston et al., 2003; Kenny et al., 2004; Patston and Osborne, 2016; Sinden, 1999) experience increased severity of MPA. Neuroticism and general trait anxiety also have been identified as risk factors for MPA (Ioannou et al., 2018; Özdemir and Dalkıran, 2017). In addition, research has demonstrated that self-efficacy, hope and resilience are reliable indicators of MPA (Arbinaga, 2023; MacAfee and Comeau, 2020; Orejudo et al., 2017; Robson and Kenny, 2017). Kaleńska-Rodzaj (2020) investigated the impact of positive psychological states and emotions on MPA. However, the precise influence of psychological capital on MPA remains underexplored. Moreover, other characteristics may mediate the association between psychological capital and MPA. High self-esteem and high-quality flow experiences have been shown to be inversely related to music performance anxiety (Girgin, 2017; Otacioglu, 2016; Spahn et al., 2021). As a result, this study is the first to simultaneously introduce two variables as mediation variables—self-esteem and flow experience—which have been linked to anxiety associated with performing music (Cohen and Bodner, 2019; Girgin, 2017). The objective is to elucidate the correlation and underlying mechanism between psychological capital and MPA, thereby offering greater insight into enhancing the mental well-being of college student music performers and elevating the standard of music performance.

### 1.1 The relationship between psychological capital and music performance anxiety

*Psychological capital* (PsyCap) originated from the positive psychology movement and refers to an individual’s positive psychological condition, encompassing four dimensions: self-efficacy, optimism, hope, and resilience. These four dimensions have a mutually beneficial and cooperative effect (Luthans et al., 2007). PsyCap serves as a valuable personal asset that can effectively aid individuals in managing anxiety and stress, thereby enhancing overall well-being (Rabenu et al., 2017). With the growing focus on MPA, there has been a confirmation of the influence of good psychological states on this issue. González et al. (2018) discovered that self-efficacy is a significant predictor of MPA. Maintaining an optimistic and positive attitude can be an effective way to cope with the challenges of music performance anxiety (Osborne et al., 2014). Additionally, hope was a strong predictor of depressive and anxiety symptoms, where higher hope correlated with lower levels of depression and anxiety (Chang et al., 2019; Richardson, 2023). Farnsworth-Grodd (2012) reported that cultivating hope-oriented coping strategies can help reduce the incidence of MPA. Psychological resilience plays a crucial role in understanding how individuals or groups manage to endure and recover from significant stressors or challenges that jeopardize their functioning, development, or well-being (Denckla et al., 2020; Leipold and Greve, 2009; Ungar and Theron, 2020). Osborne (2013) demonstrated that resilience is a crucial psychological trait in mitigating the negative effects of music performance anxiety. As the first study to explore the connection between psychological capital and music performance anxiety, this research provides initial evidence and valuable insights for future efforts to improve the mental health and manage the music performance anxiety of college students studying music. According to the Conservation of Resources (COR) Theory, when individuals experience a depletion of their psychological resources and cannot acquire fresh resources, it leads to stress and tension (Diener et al., 2003; Hobfoll et al., 2018). Nevertheless, whether PsyCap impacts music performance, students’ MPA, and potential underlying mechanisms remain unclear. Hence, investigating the correlation between PsyCap and MPA is essential. This study advances previous research on music performance anxiety by establishing the association between these two variables. Specifically, it is the first to look into the relationship and underlying mechanisms between psychological capital and music performance anxiety among Chinese music majors.

### 1.2 The mediating role of self-esteem

As one of the predictors of music performance anxiety, self-esteem may mediate the relationship between psychological capital and music performance anxiety (Sickert et al., 2022; Sinden, 1999). *Self-esteem* is an individual’s comprehensive assessment of personal value and worth (Rosenberg, 1965). According to sociometric theory, having high self-esteem has a beneficial effect on mitigating the effects of stress and adverse impacts on an individual (Leary et al., 1995). Previous research has also explored the connection between self-esteem and MPA (Chan, 2011; Girgin, 2017). For instance, Liston et al. (2003) discovered that self-esteem predicts nervousness during a musical performance. Like PsyCap, self-esteem is a crucial internal asset for lowering stress levels and decreasing anxiety (Du et al., 2017). COR theory posits that inadequate resources lead to increased vulnerability, heightened psychological stress, and emotional distress.

Conversely, sufficient resources enhance coping capacities and foster a greater sense of self-worth and efficacy (Hobfoll, 1989). Moreover, self-esteem positively correlates with PsyCap across all dimensions (Gujar and Ali, 2019). Interventions based on PsyCap have been proposed to raise self-esteem. Li et al. (2023) reported a direct relationship between high-quality PsyCap and the anxiety levels of college students who have experienced being left behind. Additionally, self-esteem acts as a mediator between these two factors. Thus, considering the aforementioned theoretical connections, we postulated that self-esteem could mediate the cross-sectional association between PsyCap and MPA.

### 1.3 The mediating role of flow experience

Flow experience, as a concept in positive psychology, refers to a mental state characterized by intense focus, diminished self-awareness, and mastery over one’s surroundings. This condition is achieved when an individual fully engages in an activity with complete dedication (Csikszentmihalyi, 1991, 2000, 2020). Jackson and Csikszentmihalyi (1999) outlined nine critical attributes of flow experience: a harmonious balance between challenge and skill, integration of behavior and consciousness, a defined objective, complete dedication, unambiguous feedback, diminished self-awareness, altered perception of time, paradoxes of control, and self-directed experience. Flow experiences are frequently linked to peak performance, and exceptional flow contributes to developing contentment and self-assurance (Habe et al., 2019; Stamatelopoulou et al., 2018). Additionally, flow experiences of inferior quality are frequently associated with feelings of anxiety and depression. Recent empirical research has discovered that the flow experience could significantly alleviate MPA. Sinnamon et al. (2012) reported that experiencing high-quality flow decreased music performance anxiety and enhanced performance quality.

In summary, strong flow experiences and lower music performance anxiety may be correlated among college students studying music performance. Positive emotions initiate an upward cycle by enhancing personal resources and increasing overall coping strategies, as suggested by resource conservation theory and positive emotion expanding and building theory (Fredrickson and Joiner, 2002). The flow experience also indicates that organizational and human resources are spiraling upward (Salanova et al., 2006). Earlier studies also provide evidence for the correlation between PsyCap and flow experience. PsyCap is positively correlated with flow experience. It can predict the occurrence of flow experience, according to research by Kawalya et al. (2019). Research has shown that flow is one of the key indicators of autonomous personality traits, with individuals who exhibit positive traits and high autonomy being more likely to experience elevated levels of flow (Asakawa, 2010; Tse et al., 2020; Tse et al., 2021). These personality traits include higher levels of autonomous motivation, self-regulation, and self-control (Kuhnle et al., 2012; Taylor et al., 2006; Wan et al., 2020). Flow has significant potential to facilitate personal growth. Furthermore, flow experiences are considered one of the precursors to self-esteem (Wells, 1988). Flow is inversely related to low self-esteem (Adlai-Gail, 1995; Csikszentmihalyi et al., 2005). Nonetheless, previous studies propose that flow experiences might be an outcome of self-esteem (Choi and Kim, 2013; Hong et al., 2019; Kim and Park, 2018). Greater self-esteem can elevate the intensity of flow (Khang et al., 2013). Mao et al. (2020) underscoring the potential of self-esteem as a precursor in cultivating an individual’s flow. Thus, drawing from the aforementioned theoretical foundation, we propose that a correlation exists between flow experience and the interplay of psychological capital and music performance anxiety in college students pursuing a major in music performance. Simultaneously, a direct relationship exists between one’s level of self-esteem and the state of flow.

### 1.4 The present study

**Figure 1:**
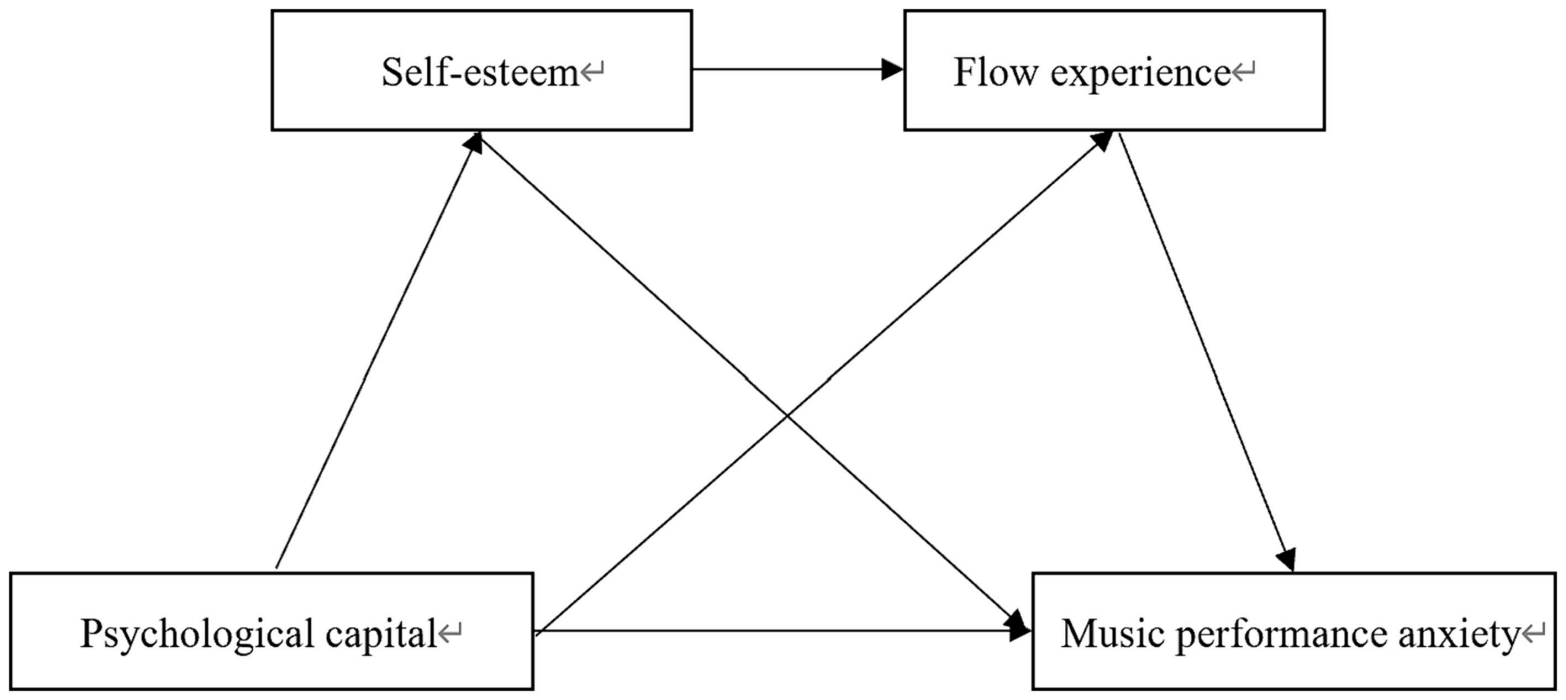
Research hypothesis model.

While past studies have noted a connection between PsyCap variables and MPA, it remains uncertain whether a direct relationship exists between PsyCap and MPA. Furthermore, prior studies have demonstrated a clear correlation between flow experience and self-esteem with PsyCap and MPA. The connection between PsyCap and MPA regarding flow experience and self-esteem remains uncertain. Consequently, drawing from the literature review above, we have formulated three hypotheses (Figure 1): (1) PsyCap, self-esteem, and flow experience are strong predictors of MPA. (2) Self-esteem and flow experience mediate between PsyCap and MPA. (3) A chain reaction between PsyCap and MPA is mediated by self-esteem and flow experience: low MPA is predicted by flow experience and correlates with greater self-esteem. PsyCap positively predicts self-esteem.

## 2 Materials and methods

### 2.1 Participants

The research was carried out in December 2023. The sampling target was undergraduate students studying music performance and education at three university conservatories in eastern China. Participants were recruited through the Internet. Flow experiences frequently arise during musical endeavors, and engaging in musical activities is widely associated with profound flow experiences (Lowis, 2002). Consequently, the participants were instructed to complete an electronic questionnaire following a music performance examination. Three-hundred fifty-seven students responded to the questionnaire. Of the 357 questionnaires collected, any that were incomplete, not completed in the expected time, or had unreliable responses were excluded. Finally, 329 questionnaires were included. Of the participants, 137 (41.6%) were males and 192 (58.4%) were females. The questionnaire gathered information on participants’ gender and obtained pertinent data regarding their age, grade, and major (Appendix 1 in Supplementary material). The study underwent ethical review and received approval according to the principles of the Declaration of Helsinki. Additionally, each student who took part in the study provided informed permission.

### 2.2 Measure

#### 2.2.1 PsyCap

The original PsyCap scale, developed by Luthans et al. (2007), was primarily designed to assess the psychological capital of corporate employees and executives, which limited its broader applicability. To evaluate students’ psychological capital in this study, we used a more general and widely applicable Positive Psychological Capital Questionnaire, specifically developed for Chinese participants (Liang et al., 2018). This research employed a Chinese version of the PsyCap scale, adapted by Kuo et al. (2010) based on Luthans et al.’s (2007) original scale. The adapted version consists of 26 items distributed across four dimensions: self-efficacy (7 items), resilience (7 items), hope (6 items), and optimism (6 items). Notably, compared to the original scale, this version includes two additional items: “I believe that society as a whole is benevolent” under the optimism dimension, and “Many people appreciate my talents” under the self-efficacy dimension. Additionally, we employed an adapted version of the scale to better accommodate the cultural context and the specific population, ensuring that responses are more accurate and meaningful. Assign scores using a 7-point Likert scale, with 1 = *highly inconsistent* and 7 = *highly consistent*. Items 8, 10, 12, 14, and 25 are reverse-scored. As the total scale score increases, PsyCap is greater. This scale has demonstrated greater reliability in Chinese samples and has been extensively utilized in prior investigations involving Chinese participants (Jiang, 2021; Wang et al., 2021; Zhang et al., 2022). The Cronbach’s alpha in this study was 0.947.

#### 2.2.2 Self-esteem

The Chinese version of Rosenberg’s Self-Esteem Scale (Rosenberg, 1965) was utilized to assess self-esteem (Shen and Cai, 2008). Prior research have employed this scale to assess self-esteem in Chinese participants (Cai et al., 2021; Zhao et al., 2021). The form comprises 10 questions, such as “Overall, I am satisfied with myself.” These questions are divided into two dimensions: positive phrasing and negative wording. The measure employs a four-point continuum, where 1 = *very low consistency* and 4 = *very high consistency*. Higher scale scores correspond to elevated levels of self-esteem. Of the above items, 3, 5, 8, 9, and 10 are typically reverse-scored. However, item 8 was not reverse-scored in this study due to cultural differences between China and Western countries (Pan et al., 2018). Cronbach’s alpha in this study was 0.886.

#### 2.2.3 Flow experience

The scale in question is a nine-dimensional scale developed by Jackson et al. (2001) derived from the nine components of the flow model proposed by Csikszentmihalyi et al. (2014). These components include challenge-skill balance, action-awareness merging, clear goals, unambiguous feedback, a sense of control, task concentration, loss of self-consciousness, time transformation, and autotelic experience. The scale comprises 36 items. A sample question is, “I frequently experience a sense of time passing swiftly.” The measure is evaluated using a five-point Likert scale from 1 (*strong disagreement*) to 5 (*strong agreement*). Cronbach’s alpha for this study is 0.956.

#### 2.2.4 MPA

MPA was measured using the K-MPAI scale developed by Kenny et al. (2004). The inventory comprises 26 items categorized into three dimensions: early vulnerability, generalized vulnerability, and concerns about performance (e.g., “I frequently experience a sense of personal worthlessness”). The rating for each item is measured on a 7-point scale, ranging from 0 (showing significant disagreement) to 6 (representing strong agreement). A higher overall score reflects a greater level of MPA. Utilizing translation software, we translated the MPA scale into Chinese. The translation underwent a review and revision process by the translator and was discussed with the first author. After addressing minor language ambiguities, the finalized version was distributed to the participants. In the present sample, Cronbach’s alpha of this scale was 0.951.

### 2.3 Data analysis

Initially, SPSS 26.0 was employed to perform Pearson correlation analysis to assess the association between the variables. Furthermore, we employed AMOS24.0 software to perform structural equation modeling and evaluate the adequacy of the model. Ultimately, the bootstrapping estimation process was employed, utilizing 5,000 bootstrap samples, to thoroughly analyze the structural equation model and determine the significance of the mediation effect.

## 3 Results

### 3.1 Correlation analysis

Table 1 presents the descriptive data, including the mean and standard deviation (SD) and the correlations among PsyCap, self-esteem, flow experience, and MPA. A substantial correlation was found between all variables in the expected directions (*p* < 0.001). The correlations between MPA and other variables are negative, indicating significant inverse associations. Conversely, the correlations between PsyCap, Self-esteem, and Flow experience are positive, indicating direct relationships. A strong positive association is expected to exist between them.

**Table 1:** Descriptive statistics and zero-order correlations for all measures.

Measure	*M*	SD	1	2	3	4
1. PsyCap	4.10	0.78	1			
2. Self-esteem	2.36	0.66	0.411**	1		
3. Flow experience	3.30	0.65	0.515**	0.398**	1	
4. MPA	3.88	0.99	−0.553**	−0.499**	−0.557**	1

Psychological capital = PsyCap; Music performance anxiety = MPA. SD = standard deviations. **p < 0.001.

### 3.2 Structural equation modeling

**Figure 2:**
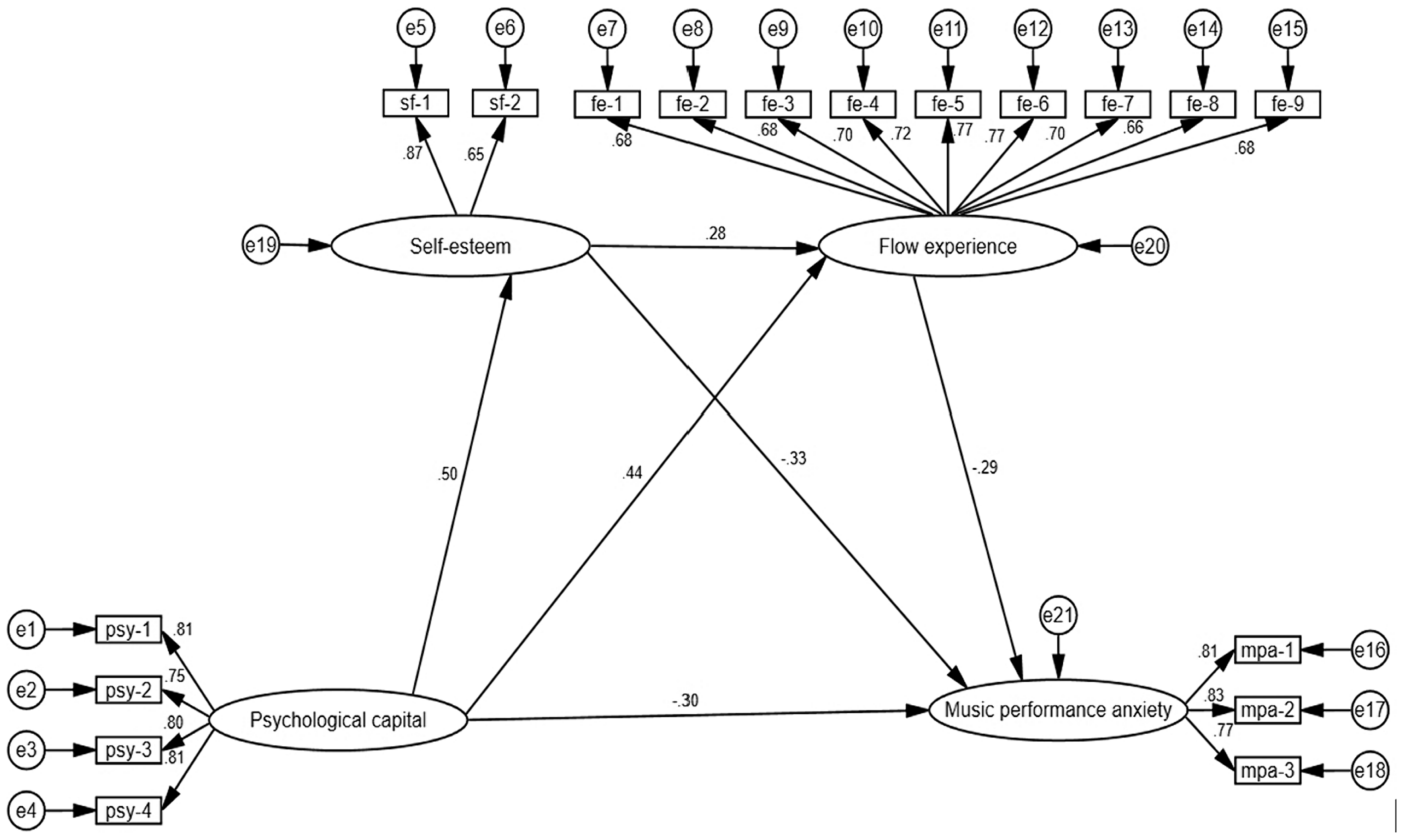
Structural equation model (*N* = 329).

To further validate the association between each variable and assess the study hypothesis, we employed AMOS 24.0 to build a structural equation model for additional examination. Figure 2 depicts the intricate path model that holds significant influence. The indicators of four latent variables are comprised of the dimensions of each scale, including an independent variable called psychological capital, which has four indicators: psy-1, psy-2, psy-3, and psy-4. The dependent variable, music performance anxiety, is measured using three indicators: mpa-1, mpa-2, and mpa-3. A mediating variable, self-esteem, is measured by two indicators: sf-1 and sf-2. Furthermore, the nine indicators in the second mediating variable, Flow experience, are represented by fe-1, fe-2, fe-3, fe-4, fe-5, fe-6, fe-7, fe-8, and fe-9. The confirmatory factor analysis (CFA) results demonstrate that the measurement model exhibits a satisfactory match to the data: χ^2^/df = 1.282; RMSEA = 0.029; SRMR = 0.033; CFI = 0.987; TLI = 0.985; GFI = 0.949; NFI = 0.945; χ^2^/df < 3; RMSEA\SRMR<0.05; CFI, TLI, GFI, NFI > 0.9. All factor loadings of the indicators on the latent variables are statistically significant (*p* < 0.001), suggesting that most fitting indicators meet high standards and the model demonstrates a strong fit.

Furthermore, the standardized path coefficient results are shown for PsyCap, self-esteem, flow experience, and MPA (Table 2). The path coefficient between PsyCap and MPA is statistically significant (β = −0.301, *p* < 0.001), suggesting that PsyCap has a strong negative relationship with the MPA of music major college students. The path coefficients from psychological capital to self-esteem (β = 0.504, *p* < 0.001) and from self-esteem to MPA (β = −0.325, *p* < 0.001) are statistically significant. This result indicates that self-esteem acts as a mediator between PsyCap and MPA, with self-esteem exerting a substantial negative influence on music performance anxiety. The path coefficient from PsyCap to flow experience (β = 0.441, *p* < 0.001) and from flow experience to MPA (β = −0.288, *p* < 0.001) is statistically significant. These values suggest that flow experience acts as a mediator between PsyCap and music performance anxiety and that flow experience has a negative influence on MPA. The path coefficient of PsyCap→self-esteem→flow experience→MPA (β = 0.277, *p* < 0.001) is statistically significant, suggesting that self-esteem and flow experience act as mediators between PsyCap and MPA in a sequential manner. Thus, all hypotheses have been confirmed.

**Table 2:** Mediating effect analysis.

Path	Estimate	S.E.	C.R.	*p*	STD. Estimate
Self-esteem←PsyCap	0.447	0.057	7.8	***	0.504
Flow experience←Self-esteem	0.25	0.066	3.766	***	0.277
Flow experience←PsyCap	0.352	0.057	6.156	***	0.441
MPA←PsyCap	−0.395	0.09	−4.38	***	−0.301
MPA←Self-esteem	−0.48	0.11	−4.348	***	−0.325
MPA←Flow experience	−0.472	0.112	−4.219	***	−0.288

Psychological capital = PsyCap; Music performance anxiety = MPA. ***p < 0.001.

### 3.3 Mediation effect assessment

The study employed the bootstrap estimation approach in AMOS to examine the mediating effects of self-esteem and flow experience on the association between PsyCap and MPA (Table 3). The mediating guide’s 95% confidence interval (CI) effect is determined using 5,000 sample iterations, and the mediating effect test is performed. The analysis findings indicate a significant overall impact of PsyCap on MPA, with a beta coefficient of −0.331 and a 95% confidence interval of [−0.434, −0.25], *p* < 0.001. When considering the direct effect alone, the impact of PsyCap on MPA is −0.301. However, when considering both the direct and indirect effects, the full impact of PsyCap on MPA is −0.632. The study found that PsyCap, mediated by self-esteem and flow experience, had a substantial indirect effect on MPA. The regression analysis showed that self-esteem had a negative coefficient of −0.164 (95% CI [−0.287, −0.083], *p* < 0.001), indicating a significant relationship. Similarly, flow experience had a negative coefficient of −0.127 (95% CI [−0.029, −0.042], *p* = 0.003), again indicating a significant relationship. Thus, Hypothesis 2 has been confirmed. Furthermore, the magnitude of the mediating effect generated by self-esteem and flow experience is (*B* = 0.04, BC 95% CI [−0.083, −0.018], *p* = 0.002). Hypothesis 3 has been confirmed. To summarize, the mediating effect explains 52.37% of the overall effect, demonstrating that PsyCap significantly impacts MPA by enhancing Self-esteem and facilitating flow experience.

**Table 3:** Bootstrap analyses of the magnitude and statistical significance of indirect effects.

Model pathways	Estimated	*p*	95%CI	
			Lower	Upper
PsyCap→Self-esteem→Flow experience→MPA	−0.04	0.002	−0.083	−0.018
PsyCap→Self-esteem→MPA	−0.164	<0.001	−0.287	−0.083
PsyCap→Flow experience→MPA	−0.127	0.003	−0.229	−0.042
PsyCap→Self-esteem/Flow experience→MPA (total indirect effect)	−0.331	<0.001	−0.434	−0.25

Psychological capital = PsyCap; Music performance anxiety = MPA.

## 4 Discussion

This study is grounded in research on the correlation between psychological capital and MPA, drawing from COR theory, flow, and expansion theories, positive emotion construction, and practical applications. It posits a strong association between psychological capital and MPA and employs empirical research to conduct statistical tests. The theoretical support for the positive impact of psychological capital dimensions, such as self-efficacy, on MPA, has been established (Egilmez, 2015). Nevertheless, further investigation is required to understand the fundamental psychological processes involved in both cases. Furthermore, this study also investigated the role of self-esteem and flow experience as mediators in the relationship above. As predicted, the findings demonstrate that psychological capital substantially reduces MPA directly or through the two underlying factors of self-esteem and flow experience. The study primarily aimed to comprehend the mechanisms underlying MPA to provide relief for music majors.

The connection between psychological capital and MPA is the foundation for further examination of their mediating effect. The findings indicate that psychological capital can forecast MPA, thus confirming Hypothesis 1. This study is among the few that examine the connection between PsyCap and MPA. Previous studies have not investigated this relationship empirically. Prior research has substantiated that certain aspects of PsyCap, such as self-efficacy and resilience, can predict MPA (Braden et al., 2015; Ritchie and Williamon, 2012).

Nevertheless, prior research has not empirically investigated the correlation between psychological capital and MPA. Furthermore, evidence supports the association between PsyCap and trait anxiety, including specific types like test anxiety (Gordani and Sadeghzadeh, 2023). In addition, these study results are supported by theoretical evidence. This study expands upon the current body of research by showing that PsyCap can predict MPA and has a beneficial influence on MPA.

Additionally, the study investigated the internal mechanisms through which self-esteem comes before flow experiences. Previous studies on MPA generally did not take into account mediating factors. Prior studies have substantiated that self-esteem is a reliable predictor of flow experience (Tomczak and Nowak, 2019). Consistent with expectations, the findings support Hypothesis 2, specifically indicating that self-esteem and flow experience partially mediate the association between psychological capital and MPA. These findings align with previous studies that have explored the relationships between psychological capital, self-esteem, flow, or MPA, though not all of these variables were examined together in those studies (Kaleńska-Rodzaj, 2020; Spahn et al., 2021). These findings suggest that college students majoring in music with elevated PsyCap, are likelier to have enhanced self-esteem and flow experiences. Consequently, this enhancement is expected to mitigate MPA. Thus, this finding may explain the high PsyCap and low MPA of students who report high levels of self-esteem and high-quality flow experiences.

The study’s findings align with the third hypothesis, which suggests that the sequence of psychological capital leading to self-esteem, flow experience, and, ultimately, MPA is indeed supported. This pathway demonstrates that self-esteem is the intermediary factor between psychological capital and flow experience, and flow experience acts as the intermediary factor between self-esteem and MPA. Self-esteem and flow experience are intermediaries between PsyCap and MPA. This discovery aligns with prior studies documenting a direct correlation between self-esteem and flow experiences (Csikszentmihalyi et al., 2005; Wu et al., 2021). These findings indicate that music major undergraduates with strong PsyCap are more likely to have higher self-esteem. This quality, in turn, is linked to experiencing more flow, associated with reducing MPA.

Overall, the study discovered that self-esteem and flow experience significantly mediate the relationship between psychological capital and MPA. Self-esteem and flow experience have a similar chain of mediating effects on PsyCap and MPA. In addition, when comparing the direct and indirect effects of psychological capital and MPA, their mediating effects contribute to over 50% of the total effect. The findings indicate that self-esteem and flow experience significantly impact PsyCap as predictors of MPA. As far as we know, this study is the first to investigate the connection between self-esteem and the mechanisms of flow experience in PsyCap and MPA. However, these findings still require comprehensive replication.

The limitations of this study must be mentioned. First, this study employed a cross-sectional design. In the future, longitudinal research can be conducted to elucidate the causality of the relationship between psychological capital, self-esteem, flow experience, and MPA. Simultaneously, all data is gathered through self-reported forms. Self-report is prone to bias. In the future, various assessment methods could be explored to mitigate the influence of data bias.

Furthermore, the study was constrained by a small sample size, and we did not assess the robustness and consistency of SEM across various age groups and genders. Ultimately, the participants in this study were exclusively limited to undergraduate music majors hailing from three universities in eastern China. Furthermore, the female representation in the sample exceeded that of males. Hence, it is necessary to conduct future experiments with larger sample sizes and ensure equal representation of genders and professions to examine the consistency of structures across various subgroups.

Notwithstanding the above limitations, this study has multiple important aspects. In theory, this study could broaden and enhance the existing MPA research foundation. Furthermore, the outcomes of the chain mediation impact of self-esteem and flow experience related to PsyCap and MPA elucidate the underlying mechanism connecting them. Furthermore, comprehending the intricate interplay among PsyCap, self-esteem, flow experience, and MPA is advantageous for developing and executing MPA intervention strategies. The findings of this study indicate that the favorable psychological attributes of PsyCap, self-esteem, and flow experience have a direct and positive influence on MPA. Practically speaking, the findings of this study indicate that music educators in higher education can create interventions focused on personal psychology to improve the positive psychological traits of music major students, thus reducing their MPA.

## Data Availability

The raw data supporting the conclusions of this article will be made available by the authors, without undue reservation.
