# Supplementary material for: Psychological capital and music performance anxiety: the mediating role of self-esteem and flow experience
Source: Front Psychol. 2024 Sep 13;15:1461235. doi: 10.3389/fpsyg.2024.1461235 (PMC11437525; doi:10.3389/fpsyg.2024.1461235)
Supplement: Supplementary file 1 [file Table_1.DOCX]

Appendix 1 Demographic information.

| Variables | Category | Frequency | Percentage（%） |
| --- | --- | --- | --- |
| Gender | Male | 137 | 41.6 |
|  | Female | 192 | 58.4 |
| Grade | Freshman | 97 | 29.5 |
|  | Sophomore | 82 | 24.9 |
|  | Junior | 72 | 21.9 |
|  | Senior | 78 | 23.7 |
| Age | 18 | 53 | 16.1 |
|  | 19 | 87 | 26.4 |
|  | 20 | 74 | 22.5 |
|  | 21 | 70 | 21.3 |
|  | 22 | 27 | 8.2 |
|  | 23 | 11 | 3.3 |
|  | 24 | 7 | 2.1 |
| Major | piano | 105 | 31.9 |
|  | vocal music | 119 | 36.2 |
|  | stringed instrument | 43 | 13.1 |
|  | brass instruments | 20 | 6.1 |
|  | woodwind instrument | 29 | 8.8 |
|  | China national musical instrument | 13 | 4 |
| Total | | 329 | 100 |
